# Assessing the Potential of Diverse Forage Mixtures to Reduce Enteric Methane Emissions In Vitro

**DOI:** 10.3390/ani11041126

**Published:** 2021-04-14

**Authors:** Cecilia Loza, Supriya Verma, Siegfried Wolffram, Andreas Susenbeth, Ralf Blank, Friedhelm Taube, Ralf Loges, Mario Hasler, Christof Kluß, Carsten Stefan Malisch

**Affiliations:** 1 Institute of Plant Production and Plant Breeding, Grass and Forage Science/Organic Agriculture, Kiel University (CAU), 24118 Kiel, Germany; sverma@gfo.uni-kiel.de (S.V.); ftaube@gfo.uni-kiel.de (F.T.); rloges@gfo.uni-kiel.de (R.L.); ckluss@gfo.uni-kiel.de (C.K.); cmalisch@gfo.uni-kiel.de (C.S.M.); 2 Institute of Animal Nutrition and Physiology, Kiel University (CAU), 24118 Kiel, Germany; wolffram@aninut.uni-kiel.de (S.W.); susenbeth@aninut.uni-kiel.de (A.S.); blank@aninut.uni-kiel.de (R.B.); 3 Grass Based Dairy Systems, Animal Production Systems Group, Wageningen University (WUR), 6705 Wageningen, The Netherlands; 4 Department of Statistics, Kiel University (CAU), 24118 Kiel, Germany; hasler@email.uni-kiel.de

**Keywords:** methane, nutraceuticals, Hohenheim gas test, plant specialized metabolites

## Abstract

**Simple Summary:**

Changes in agriculture towards simpler and more intensive systems have contributed to increased environmental problems. In temperate sown grasslands, this has resulted in ryegrass dominance, and forage legume use is limited mainly to three species: red clover, white clover and lucerne. Other dicot forages, such as *Lotus pedunculatus* and *Sanguisorba minor*, are of interest as they contain plant specialized metabolites (PSM), especially tannins, potentially reducing methane from ruminants, an important source of agricultural greenhouse gas emissions. In an in vitro study, we compared binary mixtures of perennial ryegrass with one of eight dicot species, including PSM-rich species in different proportions, to assess their potential to reduce methane production. An additional aim was to determine whether moderate additions of these forage species can be sufficient to reduce methane formation or whether the relationship is linearly dose-dependent. Results show that all dicot species studied, including the non-tannin-containing species, reduced methane production. While all plant species rich in PSM reduced methane production, they also decreased digestibility. Additionally, they did not persist in the pasture into the second year. The lowest methane emissions per digestible forage unit were obtained with chicory (*Cichorium intybus*), a promising forage herb with both agronomic and bioactive potential.

**Abstract:**

Methane emissions from ruminants are a major contributor to agricultural greenhouse gas emissions. Thus, eight different forage species were combined in binary mixtures with *Lolium perenne* in increasing proportions, in vitro, to determine their methane reduction potential in ruminants. Species were sampled in two consecutive years where possible. The aims were: a) to determine if mixtures with specific forages, particularly those rich in plant specialized metabolites (PSM), can reduce methane emissions compared to ryegrass monocultures, b) to identify whether there is a linear-dose effect relationship in methane emissions from the legume or herb addition, and c) whether these effects are maintained across sampling years. Results showed that all dicot species studied, including the non-tannin-containing species, reduced methane production. The tannin-rich species, *Sanguisorba minor* and *Lotus pedunculatus,* showed the greatest methane reduction potential of up to 33%. Due to concomitant reductions in the forage digestibility, *Cichorium intybus* yielded the lowest methane emissions per digestible forage unit. Contrary to total gas production, methane production was less predictable, with a tendency for the lowest methane production being obtained with a 67.5% share of the legume or herb partner species. Thus, linear increments in the partner species share did not result in linear changes in methane concentration. The methane reduction potential differed across sampling years, but the species ranking in methane concentration was stable.

## 1. Introduction

Simplification of agricultural systems towards crop or grass–ley monocultures and uniform, intensive production systems has contributed to several negative environmental externalities from agriculture, including soil organic carbon losses, excessive nutrient inputs into water bodies and increased greenhouse gas emissions [1]. For temperate grasslands, previous experiments and research networks have repeatedly shown potential advantages of swards based on mixtures of grasses and dicot (forb) species, particularly forage legumes and some forage herbs grass monoculture swards. These advantages occur notably under conditions with substantially reduced nutrient inputs, resulting in lower emissions to the environment, attributed mainly to improved niche utilization and facilitation effects [2]. In one example, in a 31-site plot experiment, four-species mixtures, including legumes, and receiving 150 kg, N/ha/a showed greater herbage yield than grass monocultures receiving 450 kg N/ha/a across a wide range of environments [3]. In these mixtures, legume shares of approximately 1/3 were sufficient to obtain 95% of the highest total N yield (Ntot) and exceeded the Ntot of grass monocultures by 57% [4]. The improved niche utilization also offered the potential for weed suppression, as 58% of all sites had significantly lower weed abundance than even the best performing monoculture sward [5].

Changes in grassland management regimes, with higher use intensities and shorter regrowth periods under both grazing and cutting systems, have restricted the role of legume utilization in temperate-zone temporary grasslands to three main species: red clover (*Trifolium pratense*), white clover (*Trifolium repens*), and lucerne (*Medicago sativa*). All have been subject to varietal improvement by plant breeders and are widely used globally, while other forage legumes remain less exploited [6]. This is despite the potential role for many alternative forages, legumes and also non-legume forage herbs, to increase resource use efficiency further, to reduce environmental impacts, particularly from dairy production, and to provide additional benefits for animal health due to their content of plant-specialized metabolites (PSM) [7,8,9,10]. Among these beneficial effects are anthelmintic properties, a shift in nitrogen excretion pathways from the volatile urine to the more stable form in dung, potentially higher animal productivity, and reductions in methane emissions [7]. The latter is particularly important since methane emissions from enteric fermentation are currently one of the biggest environmental burdens arising from ruminant livestock production [11], and there is considerable interest in adapting ruminant nutrition to reduce this impact. Moreover, methane emissions from enteric fermentation represent a massive loss of feed energy, and global emissions have been calculated to represent an energy loss equivalent to 144 million tons of oil [12]. Furthermore, unlike losses by ruminants via their urine and feces, these gaseous losses cannot be recycled within the agricultural system, but they contribute to the global pool of greenhouse gas emissions and thereby contribute to climate change.

Among the approaches that have the potential to reduce enteric methane emissions, the use of forages that contain PSM is particularly promising, as these may reduce methane emissions while at the same time provide high-quality forage. Of the PSM, polyphenols, and particularly tannins (both hydrolyzable tannins (HTs) and proanthocyanidins (PAs, syn. condensed tannins), have potential for use in ruminant nutrition and will be the focus of our research. PAs bind predominantly via hydrogen bonds with the rumen’s dietary protein, preventing it from enzymatic hydrolysis. Additionally, they inhibit methanogens, protozoa and generally can affect the rumen microbiota [13]. Due to these factors, they can reduce enteric methane production. The PA-protein complexes dissociate in the intestine as a result of the increased pH, thereby enabling increased amounts of utilizable protein at the duodenum (uCP), which is subsequently absorbed as amino acids. This can increase the forage nutritive value without depressing rumen fiber digestion or daily intake [9,14]. Among the PA-containing forages, the legumes birdsfoot trefoil (*Lotus corniculatus*), big trefoil (*Lotus pedunculatus*) and sainfoin (*Onobrychis viciifolia*) have all shown good potential as sources of feed for livestock [15]. However, their antimethanogenic effects have been variable in previous findings (e.g., [16,17]). The variability in the results could be attributed, at least partially, to the often insufficient characterization of the structural characteristics of the tannins [13]. Additionally, the co-presence of other PSM, such as flavanols and saponins, has been shown to interact with tannins, with potentially mutualistic or antagonistic effects [18,19]. Furthermore, the growth conditions of the tested plants varied across experiments, affecting not only the synthesis of PSM [20,21,22] but also the forage chemical composition [23], hence constituting an additional source of variability in methane emissions. Finally, many previous studies have been performed with plant extracts and purified compounds, with little applicability for grazing animals or even current indoor systems.

Several other non-legume forage species with high feeding value, such as chicory (*Cichorium intybus*) with lower CT contents, but rich in other phenolic compounds and rapidly fermentable carbohydrates, and ribwort plantain (*Plantago lanceolata*), which lacks CT, but has a high nitrogen use efficiency in dairy cows, potentially due to a favorable water-soluble carbohydrate ((WSC):N ratio) [24], also appear to be beneficial, but there have been few studies on their inclusion in diets related to methane emissions. Furthermore, and in contrast to the nitrogen-derived benefits of diverse swards, there has not yet been any study that has identified the required shares of potentially bioactive components in forage mixtures needed to obtain a desired antimethanogenic effect. Hence, the study reported in this paper aimed to provide the first comparison of specifically designed binary forage mixtures of perennial ryegrass (*Lolium perenne*) with one of eight different forage species in increasing shares and across two different years to test the following hypotheses: (a) tannin-containing forages reduce methane emissions compared to non-tannin forage species, (b) the antimethanogenic effect is not linear, and moderate additions of partner species can be sufficient to reduce methane emissions with lower reductions of digestibility, and (c) these findings will be affected by the growth conditions, but the general species-specific reduction potential is replicable across harvest years.

## 2. Materials and Methods

The in vitro analysis was performed between August and December 2019 at the laboratory of the Institute of Animal Nutrition and Physiology of Kiel University, Germany.

Eight species, including legumes and non-legume forbs (hereafter called “partner species”), were combined in binary mixtures with perennial ryegrass (*Lolium perenne*) used as the common species, in increasing proportions (25%; 37.5%; 50%; 62.5%; 75% and 100%). The *Lolium perenne* monoculture represents the treatment with 0% partner species. Table 1 lists all partner species used in the binary mixtures. Species listed only in one year (2018) were outcompeted and did not persist in the second year in the field.

### 2.1. Sample Collection

All forage species were sown in autumn 2016, in plots of approximately 2.2 ha in a diverse mixture, integrating a two-year grass-clover ley system in an organic 4-year crop rotation (2 years of grass-clover, followed by 2 years of crops: oats/winter triticale, faba beans/winter spelt, consecutively), at “Lindhof”, the experimental farm for organic production of Kiel University (54°27′56.0″ N 9°57′56.0″ E). Samples of each species for the in vitro analysis were collected from these multispecies swards before grazing in spring, in two consecutive years (2018 and 2019) when the sward was at a late vegetative growth stage. Some species (*L. corniculatus* Lotanava, *L. pedunculatus* and *S. minor*) were harvested only in 2018 due to their low persistence. A sample of approximately 300 g of fresh plant material was cut to a residual height of 4 cm. Samples were immediately frozen and stored at −28 °C. Before analysis, samples were freeze-dried to a constant weight and milled to a particle size of 1 mm (Ultra centrifugal mill, ZM200, Retsch GmbH, Haan, Germany).

### 2.2. In Vitro Incubation

Total gas and methane production of these forage mixtures were determined using the Hohenheim gas test (HGT) according to the procedure described by Menke and Steingass [25]. Each forage mixture was incubated in triplicate, and these incubations were repeated two times within an interval of one week. In addition to the samples, four blank syringes (rumen fluid without added plant material) and three replicates of hay and concentrate standards (200 ± 1 mg) were included to correct the gas production each time.

Incubations were run for 24 h with a recording of total gas production and methane production at 8 and 24 h. Subsequently, these values were adjusted to the dry matter and digestible organic matter (DOM) content of the incubated substrates. Methane concentration in the total gas was measured by an infrared methane analyzer (Pronova Analysentechnik GmbH & Co. KG, Berlin, Germany). Total gas production was obtained according to the following equation:

((V24 − V0 + V8 − Z − Gb0) × F × 200)/W(1)

where Gb is the total gas produced (mL/200 mg DM); V0, V8 and V24 is the gas volume (mL) at 0, 8 and 24 h from the beginning of the incubation time, respectively; Z is the volume of the sample without gas; Gb0 is the gas volume before the incubation; F is the average of the correction factor for hay and concentrate, and W is the weight of the sample (mg).

The rumen fluid for this analysis was obtained from four rumen-cannulated, non-lactating Jersey × German Black Pied heifers, with an average body weight of 565 ± 29 kg, belonging to the Institute of Animal Nutrition and Physiology of Kiel University. Animals were fed a ration consisting of 3 kg of grass hay and 3 kg of concentrate divided into 2 meals (7 a.m. and 4 p.m.). Approximately 750 mL of rumen fluid was collected before the morning feeding for each incubation run. The fluid was subsequently filtered through a cheese-cloth and transferred into a prewarmed thermos flask, and immediately transported to the laboratory. The preparation of incubation media, filling of syringes and all additional procedures were done according to the protocol of Menke and Steingass [19], and 200 mg (±1 mg) of each replicate were weighed individually and filled into 100 mL fermentation syringes with 30 mL of rumen fluid, for incubation.

### 2.3. Sample Analysis

#### 2.3.1. Analysis of the Chemical Composition

All forage samples used in the in vitro incubation were analyzed using near-infrared reflectance spectroscopy (NIRS) with a NIRSystems 5000 monochromator (FOSS, Silver Spring, MD, USA). A mathematical evaluation of the spectra was performed using the Modified Partial least-squares method (WinISI software version 3, Infrasoft International, USA). Calibration and validation were based on sample subsets of perennial ryegrass, legumes and forage herb species, representing the whole spectral and chemical variability.

The following analyses of the subset samples were performed as follows: N concentration was directly determined with an elemental analyzer (Vario Max CN, Elementar Analysensysteme, Hanau, Germany); crude protein content (CP) was calculated from the N content (CP = N × 6.25). The concentrations of neutral detergent fiber (assayed with heat-stable amylase (aNDF)) and acid detergent fiber (ADF) were analyzed using a fiber analyzer Ankom A2000 (Ankom Technology, Macedon, NY, USA). ADF values are expressed exclusive of residual ash (ADFom). Ash (A) was determined by combustion in a muffle furnace (24 h at 550 °C). Digestibility and metabolizable energy (ME) content of herbage samples were determined using the in vitro cellulase technique developed by De Boever et al. [26]. The percentage of digestible organic matter (DOM) was calculated using the enzymatic soluble organic matter (ELOS) and the enzymatic insoluble organic matter (EULOS).

The following equations were used:

DOM (%) = 100 (940 − A − 0.62 EULOS − 0.000221 EULOS^2^)/(1000 − A) (2)

EULOS (g/kg DM) = 1000 − A − ELOS(3)

ME (MJ/kg DM) = 5.51 + 0.00828 ELOS − 0.00511 A + 0.02507 CL − 0.00392 ADFom(4)

where: A, CL (crude fat), ELOS, EULOS are expressed in g/kg DM.

The statistical key figures of the NIRS calibration and validation are given in Table A1.

#### 2.3.2. Analysis of Tannin Composition

Tannin extraction and sample preparation were done as described by Malisch et al. [27]. Briefly, forage samples were freeze-dried and subsequently ball-milled using a MM 200 mill (Retsch Technology GmbH, Haan, Germany). Plant samples (20 mg) were weighed into 2 mL Eppendorf tubes and stored at −80 °C until further analysis. For extraction, 1.4 mL of acetone/H_2_O (80:20, *v/v*) was added into the Eppendorf tubes, which were then shaken for 15 min in a vortex shaker. The plant and solvent mixture was macerated in a refrigerator overnight to enhance the extraction efficiency of the, especially large PAs. While macerating in the refrigerator, the tubes were shaken in a planar shaker before centrifuging at 9000 g and decanting the solvent. Solvent samples were concentrated for approximately 2 h in an Eppendorf concentrator plus (Eppendorf AG, Hamburg, Germany) to remove the acetone, and the plant residues were extracted with 1.4 mL of new acetone and H_2_O solution (80:20, *v/v*) for an additional 3 h. The two extracts were then combined and concentrated into the water phase. The extracts were then frozen and freeze-dried (Beta 1–8 LD plus, Martin Christ GmbH, Osterode, Germany) overnight and stored at −20 °C. Before injection in the ultra-performance liquid chromatography–tandem mass spectrometer (UPLC-MS/MS), samples were dissolved in 1 mL of ultrapure water, shaken for 10 min, filtered with 0.2 μm PTFE syringe filters (VWR International, Radnor, PA, USA), and diluted 2-fold with ultrapure water.

After milling, partner species were homogenized, and a subsample was taken for analysis via UPLC-MS. The UPLC-MS/MS analysis of all forage samples was conducted according to Engström et al. [28]. Therefore, the setup was identical to the Acquity UPLC system (Waters Corp., Milford, MA, USA), connected to a Xevo TQ triple-quadrupole mass spectrometer (Waters Corp., Milford, MA, USA) with electrospray ionization. For the UPLC system, a 100 mm × 2.1 mm i.d.,1.7 μm, Acquity UPLC BEH phenyl column was utilized (Waters Corp., Wexford, Ireland). Quantification was made via a diode array detector and the Xevo TQ mass spectrometer, using multiple reaction monitoring (MRM) methods. Before each run, to determine the system’s performance, a flavonoid mix stock solution was injected twice. Additionally, before and after every 10 samples, 5 samples of a catechin stock solution (1.0 μg mL^−1^) were run to determine the stability of the system’s ionization efficiency for polyphenols throughout the analysis and to correct the measured concentrations accordingly.

### 2.4. Statistical Analysis

The effect of the differing partner species on methane and total gas emissions was analyzed with the statistical software R Core Team [29] in three different approaches:

(1) A linear mixed model was used [30], with the partner species, the sampling year and the partner proportion, and their interactions (two- and threefold) as fixed factors, while partner species nested in the date of analysis was the random factor. Residuals were assumed to be normally distributed and heteroscedastic, based on graphical residual analysis, and the heteroscedasticity was accounted for. Subsequently, multiple contrast tests [31] were conducted to compare the different levels of the influence factors. With Y being the response variable, the model was:

Y_ijlm_ ~ a_i + (ab)_ij_ + c_k_ + (cd)_kl_ + e_ijklm_ (5)

where: Y_ijlm_ is the response variable of the mth piston of each partner share i in the combination of partner species and sampling year j, at each individual measurement date k for each species l, respectively. The levels of the respective fixed factors were: i = 1, …, 6 (partner share); j = 1, …, 17 (combination of partner species and sampling year); while the random terms had the levels; k = 1, …, 14 (date of in vitro analysis); l = 1, …, 10 (partner species); and the error term included additionally: m = 1, 2, 3 (piston replicate).

(2) To analyze the change in methane and total gas formation as a slope for each individual species, the model from I) was modified to now include the proportion of the partner species as a quantitative factor, together with a pseudo factor representing the combination of the actual factors partner species and sampling year. The pseudo factor was necessary, as the actual factors were not fully orthogonal due to the lack of three species in the 2019 sampling. The partner species nested in the analysis date was again the random factor, and residuals were again assumed to be normally distributed and heteroscedastic based on graphical residual analysis, and the heteroscedasticity was accounted for. Based on this model, Pseudo R^2^ (coefficient of determination) values for each species were calculated according to Nakagawa and Schielzeth [32]. Slopes were tested to be significantly different from zero to identify which species would result in significant increases or decreases in methane or total gas emissions with increasing partner shares. Additionally, due to many species, the grand mean was used to determine which species would perform better (or worse) than the average.

(3) Additionally, covariates with prediction potential for changes in CH_4_ or total gas emissions were identified using parameters from forage quality or PSM. For this, it was tested whether a model based on these covariates would explain the emissions similarly and well, compared to the linear mixed model, with the aim of generating a formula that could predict CH_4_ emissions and digestibility, independently of the plant species. In total, 18 covariates were assessed in this approach, including the main forage quality parameters listed in Table 2, as well as the plant secondary metabolite concentrations listed in Table 3. Subsequently, an analysis of covariance was performed based on the model described in Equation (5), adding covariates stepwise using a forward selection, based on Akaike information criterion (AIC) values [33]. Finally, covariates with a variance inflation factor (VIF) of 5 or greater were removed.

## 3. Results

The results are presented below as follows: (i) the measured values of the in vitro analysis and for methane and total gas are presented for the individual mixtures, including the pair-wise comparisons between the increasing legume/herb shares. With this, the observed variability across the mixtures can be evaluated and the potential linearity of dose–response effects assessed. Additionally, the analysis of covariance (ANCOVA) is presented to identify whether forage characteristics alone suffice to explain methane and total gas production. This is followed by (ii) the modeled slopes for each individual species, which are tested against the grand mean to identify their general methane reduction potential and the relative change in digestibility. Finally, the model performance against the measured values is presented.

### 3.1. Measured Parameters

#### 3.1.1. Forage Quality Was High for All Species and across Both Years

The energy concentration calculated from NIRS-based forage quality parameters was validated against the energy concentration as derived from total gas formation in the HGT (Table 2). The general coefficient of determination was R^2^ = 0.44. When excluding the forage species with the highest tannin concentrations, *L. pedunculatus* and *S. minor*, this increased to R^2^ = 0.70. The best coefficient of determination was achieved in the group of non-tannin-containing forage legumes and herbs with R^2^ = 0.88. Independent of the estimation method, the energy concentration was still high for all species throughout both years, despite general decrements for legumes and herbs in the second year. The species with the greatest energy content as ME in 2018, according to the total gas formation, was *L. perenne*, with 11.6 MJ ME/kg DM, followed by *L. corniculatus* Leo, *C. carvi* and *C. intybus*, all of which had energy concentrations greater than 11.0 MJ ME/kg DM. The OM digestibility (DOM) was greatest in *C. carvi*, *C. intybus* and *P. lanceolata* with 920 g/kg DM on average. *Sanguisorba minor* and *L. corniculatus Leo* had lower mean values of DOM (on average 870 g/kg DM), while *L. pedunculatus* was the species with the least DOM (800 g/kg DM, −9% when compared to the average of all partner species). *Carum carvi* showed exceptionally low CP contents (67 g/kg DM, ~55% less than the average of all partner species), while *T. repens* and *T. pratense*, the most common forage legumes, showed the greatest CP contents (211 g/kg DM, on average).

In 2019, when compared to 2018, the energy concentration of *L. perenne* increased even further and reached 12.1 MJ/NEL kg DM and a DOM of 910 g/kg DM. Almost all partner species showed a decrease in energy content (−4% NEL, on average), and *T. repens* was the only species that exceeded 11.0 MJ NEL/kg DM. In addition, the DOM of partner species generally decreased (−5%, on average) as a consequence of a greater NDF concentration (+21%; Table 2).

#### 3.1.2. Plant Secondary Metabolites Varied across Species with *L. pedunculatus* and *S. minor* Showing Highest Tannin Concentrations

In 2018, the greatest concentration of polyphenols was observed in *S. minor* (47.6 mg/g DM; Table 3), of, which 86% were hydrolyzable tannins, and the rest were flavonols. The second-highest polyphenolic concentrations were measured in *L. pedunculatus*, with 20.8 mg/g DM, of which 88% were proanthocyanidins (PA), with a prodelphinidin (PD) share of 79%. The cultivar *L. corniculatus* Lotanava showed greater shares of total polyphenols, as well as total tannins, compared to *L. corniculatus* Leo, with CT concentrations of 4.1 and 1.7 mg/g DM, respectively. Both *Trifolium* spp. showed greater shares of flavonols (89% and 66% of total polyphenols for *T. pratense* and *T. repens*, respectively), but almost no proanthocyanidins (0.3 and 1.8 mg/g DM for *T. pratense* and *T. repens*, respectively). The lowest polyphenol concentrations were generally observed in *P. lanceolata*.

As the species with the greatest tannins content in 2018 (i.e., *L. pedunculatus* and *S. minor*) could not be sampled for analysis in 2019, *L. corniculatus* Leo showed the greatest tannins contents compared to the other species in 2019. However, these concentrations were substantially lower at a total tannin concentration of 2 mg/g DM. The highest polyphenolic concentrations were measured in *C. carvi* with 5.9 mg/g DM. While the metabolic profile in *L. corniculatus* Leo and *P. lanceolata* was stable between 2018 and 2019, the polyphenolic concentrations in *C. carvi*, *C. intybus* and *T. repens* decreased from 2018 to the 2019 sampling, yet not uniformly, as larger declines were observed in *T. repens* and *C. intybus* with 55% less total polyphenols, each, and no measurable PA concentration in *T. repens*.

#### 3.1.3. Total Gas Production Was Generally Linked to the Mixture Composition Linearly, While Changes in Methane Were Less Predictable

In 2018, total gas production was generally dose-dependent and decreased as the proportion of the partner species increased (Table 4 and Table 5). Accordingly, the largest reduction when compared with *L. perenne* was always obtained with the pure partner species. The largest reduction was 32%, which was obtained with *S. minor* (Table 5). Of these species, particularly *S. minor* and *L. pedunculatus* showed high coefficients of determination with R^2^ = 0.74 and R^2^ = 0.72, respectively (Table 6). In contrast, methane formation was not dose-dependent, and the largest methane reduction was not achieved with pure partner species but was obtained at the ratio of 67.5% partner species with 32.5% *L. perenne*, with emissions tending to increase again with a proportion of partner species in the ratio. However, the differences across partner shares were generally not significant within any species, and only in *L. pedunculatus*, *S. minor* and *P. lanceolata* were these lowest emissions different from even the highest emissions in the pair-wise comparison (Table 4 and Table 5). In addition, due to the nonlinear relationships of methane formation per increasing partner share, coefficients of determination were generally low and only significant for *L. pedunculatus* and *S. minor* (Table 6).

In 2019, with the exception of *C. intybus*, there was again a dose-dependent effect, and this obtained the lowest total gas formation at 100% partner share. However, in the absence of the species with the highest tannin concentrations, it was *L. corniculatus* Leo that now obtained the lowest total gas formation with reductions of 21% compared to *L. perenne* (Table 4) and *L. corniculatus* Leo also showed the highest coefficient of determination (R^2^ = 0.74) (Table 6). With regard to methane formation, while there was again no dose-dependent effect in 2019, there also was no trend of lowest methane formations occurring at 67.5% of partner species. In fact, there was no discernable trend at all (Table 4). Accordingly, the highest coefficients of determination were obtained with *C. intybus* and *L. corniculatus* Leo, with R^2^ = 0.34 and R^2^ = 0.39, respectively (Table 6).

The methane concentration (i.e., methane/total gas ratio) expresses how methane changes compared to total gas, which is a proxy of digestibility. Here, the coefficients of determination were again generally higher in 2018, compared to 2019 (Table 6). However, *T. pratense* and *T. repens* were the species with the highest coefficients of determination in both years.

#### 3.1.4. Covariates Did Not Improve the Model’s Ability to Predict Rumen Fermentation Parameters

The ANCOVA did not show any covariates that improved the basic model with the fixed factors partner proportion and the interaction of partner proportion, and a pseudo factor derived from partner species and sampling year, for either the total gas or methane production. For total gas production per g DOM, only the interaction of partner proportion with the pseudo factor of partner species and harvest year was significant (F_14,416_ = 31.6, *p* ≤ 0.001), while the partner proportion alone was not significant (F_1,416_ = 0.7, *p* = 0.39; results not shown). The marginal R^2^ for the final model was 0.70, and the conditional R^2^ was 0.84, indicating that the fixed factors were able to explain most of the variability.

With regard to the methane production per g DOM, again, only the interaction of the partner proportion with the pseudo factor derived from partner species and sampling year (F_14,413_ = 43.6, *p* ≤ 0.001) was significant, with the partner proportion alone being less relevant (F_1,413_ = 3.81, *p* ≤ 0.05; results not shown). The marginal R^2^ for the final model was 0.42, while the conditional R^2^ was 0.91, indicating that the fixed factors explained less than half of the observed variation. None of the covariates were able to improve the quality of the model.

For the methane concentration in the total gas, several covariates were significant and improved the model’s quality. Accordingly, a measured impact was determined for NDF content (F_1,423_ = 16.7, *p* ≤ 0.001), hydrolyzable tannins (F_1,423_ = 63.6, *p* ≤ 0.001), total myricetins (F_1,423_ = 9.7, *p* ≤ 0.001), total quinic acid (F_1,423_ = 266.9, *p* ≤ 0.001), total kaempferols (F_1,423_ = 28.2, *p* ≤ 0.001) and total prodelphinidins (F_1,423_ = 63.6, *p* ≤ 0.001; results not shown). Despite the large number of significant covariates, the marginal R^2^ for the final model was 0.13, while the conditional R^2^ was 0.98, indicating a low predictive capacity of the fixed factors. Of these factors, NDF, total myricetins and total kaempferols had a positive effect on the methane concentration, while hydrolyzable tannins, total quinic acid and total prodelphinidins had negative relationships (results not shown).

### 3.2. Modeled Fermentation Parameters

#### 3.2.1. Tannin-Containing Species Reduce Methane Formation Most, but Do Not Provide the Lowest Methane Concentrations

**Figure 1:**
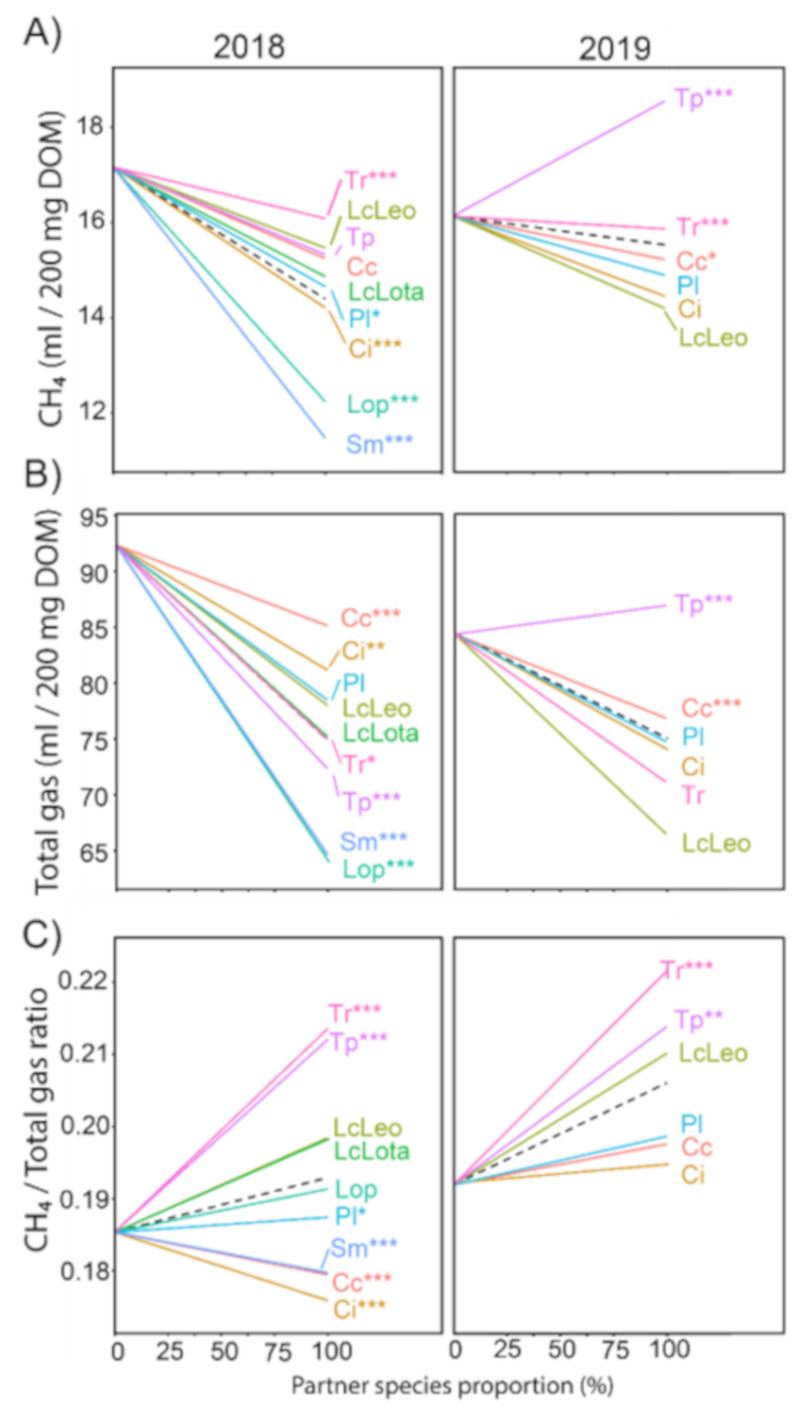
(**A**) Methane production (mL/200 mg DOM), (**B**) total gas production (mL/200 mg DOM) and (**C**) methane/total gas ratio, after 24 h-rumen fermentation of the selected forage species harvested in 2018 and 2019, mixed in increasing proportions with perennial ryegrass (Significance levels are: *** *p* ≤ 0.001, ** *p* ≤ 0.01, * *p* ≤ 0.05 and ‘ ‘*p* > 0.05). The dashed line corresponds to the Grand Mean. Abbreviations are as follows (in alphabetical order): Cc: *Carum carvi,* Ci: *Cichorium intybus*, LcLeo: *Lotus corniculatus* cv. Leo, LcLota: *Lotus corniculatus* cv. Lotanava, LoP: *Lotus pedunculatus*, Pl: *Plantago lanceolata*, Sm: *Sanguisorba minor*, Tp: *Trifolium pratense*, Tr: *Trifolium repens.*

There was generally a decrease in methane production with increasing shares of partner species (Figure 1). In 2018, the greatest methane reductions (*p* ≤ 0.001) were predicted by the model for the species with the highest tannin concentrations, *L. pedunculatus* and *S. minor*, with slopes of −4.9 and −5.7, respectively (Figure 1). The third highest reductions (*p* ≤ 0.001) were obtained with *C. intybus*, with a slope of −3.0, which was the only other species, which had emission reductions significantly lower than the grand mean of all partner species (*p* ≤ 0.001). However, for *L. pedunculatus* and *S. minor,* the model also predicted the highest reductions (*p* ≤ 0.001) in total gas, with slopes of −28.0 and −27.7, respectively. Contrary to that, *C. carvi* and *C. intybus* reduced total gas formation significantly less than the grand mean, with slopes of −7.2 (*p* ≤ 0.001) and −11.2 (*p* ≤ 0.01). As a result, the methane concentration of *L. pedunculatus* exhibited a slope of 0.005 and thus constituted a slight increment compared to that of pure *L. perenne*. Contrary to that, *S. minor* was predicted to reduce (*p* ≤ 0.001) the methane concentration, but only slightly, with a slope of −0.005. However, the strongest reduction was achieved with *C. intybus*, with a slope of −0.009, thus being almost twice the slope of the best tannin-containing species.

In 2019, in the absence of the species with the highest tannin concentration, the strongest methane reduction potential was predicted for *L. corniculatus* Leo, with a slope of −1.9, which was not significantly less compared to the grand mean, but was different from zero (*p* ≤ 0.001; result not shown). The second greatest reduction was predicted for *C. intybus*, with a slope of −1.7. However, when looking at total gas formation, *L. corniculatus* Leo was also predicted by the model to result in the greatest reduction of −17.9, being much higher than the slope of *C. intybus*, which was estimated to be −10.3. While both were not significantly less than the grand mean, both slopes were significantly different from 0 (*p* ≤ 0.001, result not shown). As a result, the slope of *C. intybus* for methane concentration was the lowest with 0.003, which was not significantly different from 0 (result not shown), while the methane concentration of *L. corniculatus* Leo was predicted to increase with a slope of 0.02, which was not significantly different from the grand mean (Figure 1), but was different (*p* ≤ 0.001) from 0 (result not shown). The strongest increments in methane concentration were predicted for the most widely used forage legumes, white and red clover, with slopes of 0.03, which were greater (*p* ≤ 0.001) than the grand mean and different from 0 (*p* ≤ 0.001, result not shown).

#### 3.2.2. Despite Differences in Total Gas and Methane Formation, Trends in Methane Concentration Are Stable across Both Experimental Years

Despite the fact that the most important of the tannin-containing species could not be sampled in 2019 again, six partner species were harvested in both of the two years, and their responses can be compared. Due to changes in forage quality in perennial ryegrass, the intercept of the model changed between these two years and was 17.2 and 16.1 mL/200 mg DOM for the methane formation, 92.3 and 84.4 mL/200 mg DOM for the total gas, and 0.185 and 0.191 for the methane concentrations of samples from 2018, and 2019, respectively (Figure 1). Due to the non-orthogonal design, interactions between partner species and year cannot be evaluated, yet both slopes and the order of species slopes differed between years for both methane and total gas formation. Accordingly, *L. corniculatus* Leo, which was the best of the tested treatments at reducing methane formation in 2019, was second-lowest in 2018, while *T. pratense* reduced methane emissions with a slope of −1.8, yet increased methane formation in 2019, with a slope of 2.4, both of which were significantly different from 0 (*p* ≤ 0.001, result not shown). Similarly, *T. pratense* was predicted to reduce total gas formation in 2018 with a slope of −20.0, compared to 0 (*p* ≤ 0.001), yet was predicted to have a non-significant tendency to increase with a slope of 2.6 (result is not shown). Despite that, the ranking of species for methane concentration was the same across both years regardless of the fact that *C. carvi* and *C. intybus* had not been predicted to result in negative slopes in 2019, compared to 2018. Furthermore, when testing all slopes from 2018 against those of 2019, the coefficient of determination equals R^2^ = 0.94 (result is not shown), which indicated that generally, the change in methane concentration with an increasing share of partner species remains stable across species and years.

### 3.3. Performance of Model Compared to Measured Data

**Figure 2:**
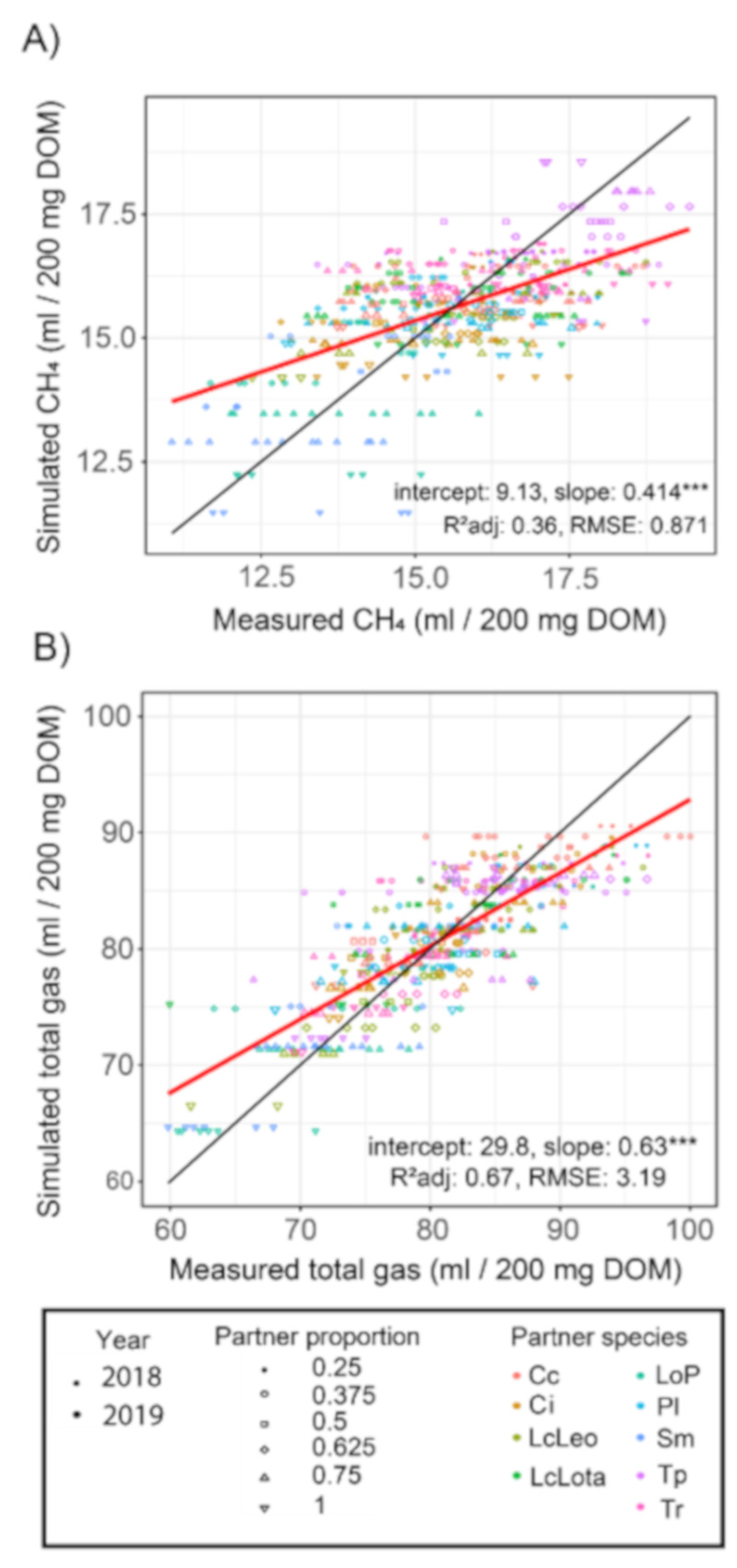
Comparison of simulated vs. measured data for methane production (**A**) and total gas production (**B**). Significance levels are: *** *p* ≤ 0.001). Abbreviations are as follows (in alphabetical order): Cc: *Carum carvi*, Ci: *Cichorium intybus*, LcLeo: *Lotus corniculatus* cv. Leo, LcLota: *Lotus corniculatus* cv. Lotanava, LoP: *Lotus pedunculatus*, Pl: *Plantago lanceolata*, Sm: *Sanguisorba minor*, Tp: *Trifolium pratense*, Tr: *Trifolium repens.*

When comparing the model performance against measured data, slopes were less than 1 for both the methane production (slope = 0.41, Figure 2A) and total gas production (slope = 0.63, Figure 2B). Both intercepts were positive, with 9.1 and 29.8 for methane production and total gas production, respectively. Thus, the model generally tended to overestimate lower emissions while underestimating higher emissions. Across both years, the general model performance was better for total gas (R^2^ = 0.67, root-mean-square error (RMSE) = 3.19) than for methane (R^2^ = 0.36, RMSE = 0.87).

## 4. Discussion

### 4.1. The Experimental Setup Was Suitable to Answer the Research Question

The lack of a linear dose-dependent reduction in measured methane emissions due to the variability between increments in the share of partner species was surprising. This variability does not appear to be a methodological error, as (a) standard errors were relatively small, indicating a reliable performance of the Hohenheim gas test, (b) all samples were mixed individually rather than as pool samples, which were then split for the replicates, making weighing mistakes unlikely, and (c) total gas production showed trends that were much more linear and also showed a much better performance of our models. Consequently, the variation in methane production suggests that methane production is much harder to predict and either require more inputs than forage quality and the main plant secondary metabolites, or several of these compounds interact with matrix effects, thereby leading to less predictable results. In the present study, it is probable that the composition of the diet fed to the donor cows (from which the rumen fluid for the in vitro test was extracted) may have had an effect on CH_4_ production, as the donor cows were fed a diet with 50% concentrate, to evaluate the performance of the different forages, with greater contents of fiber. Considering that methanogenic archaea are very sensitive to changes in the rumen pH, with their optimum range between 6.5 and 7 (achieved with diets rich in fiber), a rumen environment adapted to diets rich in concentrate, with lower pH, would limit the methanogenic activity [34]. Therefore, it is possible that CH_4_ production recorded in this experiment may have been underestimated due to this effect of the diet composition. However, it should be noted that the procedure for the performed in vitro test (HGT) is standardized, ensuring the comparability of the results. Additionally, in a review from Yáñez-Ruiz et al. [35], it is mentioned that when the rumen fluid is taken in the morning, immediately before feeding, as was the case in our experiment, the differences in the gas production profile from donors fed a grass silage:barley grain diet (80:20) or a barley straw diet were minimal. Furthermore, given the lower persistence of the PSM-rich species in the study, which results in low shares in the forage mixtures offered to the cattle, as also found in our previous in vivo study [36], the addition of these forages in high-energy diets (with greater shares of concentrate) for high yielding cows must be considered as a strategy. In this case, the use of rumen fluid from donors fed a diet rich in concentrates will make our results more comparable for these systems. Additionally, it is worth noting that the choice of *L. perenne* as the grass partner species was made according to its representativeness of the pasture-based systems. However, because of the high quality of this forage, the reduction in CH_4_ production by the additions of bioactive herbs may have been underestimated. Conversely, if the reference species had been grass with a lower content in WSC or generally a lower digestibility, then the reduction in methane emissions had likely been larger. On the other hand, Jayanegara et al. [18] highlighted that the variation in methane production per digestible OM in vivo was particularly high when the dietary tannins were low (≤20 g/kg DM), as was the case for most of the species, and especially for almost all mixtures assessed in the present study. According to Jayanegara et al. [18], this was probably due to the influence of other dietary components masking the effects of tannin at low levels, and this effect was particularly pronounced when testing the effects of PSM forages in mixtures in vitro. This is consistent with previous studies, in which the only one of these two factors were varied by using purified tannin extracts, which were added to a constant feed source. This generally provided clearer results; for example, Tiemann et al. [37] observed clear plant-specific effects (*p* ≤ 0.001) of purified tannin extracts at different levels (0, 25, 50, 75 and 100 mg/g of forage dry matter (DM)) of purified PA from four seminiferous shrub legumes, added to a grass–legume mixture). Nevertheless, these two approaches represent different practical implications, with one being the supplementation of tannins in a total mixed ration (TMR) diet, whereas the mixing of plant species represents an approach that is suitable for pasture-based forage provision. Thus, despite the larger variability, both approaches are required to identify the most suitable improvements for both systems.

Additionally, several studies have identified the incubation time to be relevant when analyzing mixtures. Accordingly, Niderkorn, Baumont, Le Morvan and Macheboeuf [10] and Robinson et al. [38] found that non-additive effects of feed mixtures on total gas production in vitro are clearer after short incubation periods of 3 h to 8 h when compared to 24 h. These findings are following Goel and Makkar [39], who reported a lack of significant non-additive effects on methane emissions after 24 h incubation. Therefore, the variation observed in measured methane production could also be attributable, at least in part, to the incubation time of 24 hours used in the present study. However, the variation during the initial hours is mainly due to the fermentation dynamics, as the fermentation of most soluble fractions and the protein synthesis occurs within the first three to five hours of incubation, while the non-soluble fraction degrades in the later hours. Thus, the 24 h are generally more representative of the overall feed performance and hence are also most frequently used when estimating the energy content of feeds [25].

Regarding the overall model performance, the model generally performed better at estimating total gas production than at estimating methane production. For methane, when comparing the modeled values against measured data, the model tended to generally overestimate lower emissions (i.e., the high partner shares) while underestimating higher methane emissions (i.e., the *L. perenne* monoculture and low partner shares). Accordingly, the model can be considered conservative in its statements and generally underestimates the partner species’ methane reduction potential. As mentioned previously, this suggests that methane is much more complex to predict than total gas, as a result of a combination of factors that may include the characteristics of the rumen fluid utilized and the NDF (and other carbohydrates) content (as a substrate for enteric fermentation). Nevertheless, independent of the reasons for the observed variability, the fact that the methane concentration was generally comparable across both years, and that also all pseudo-regressions exhibited slopes, which showed a good fit and mostly were significantly different from 0, indicates that the reduction in methane formation and in part in methane concentration of the tested species is a valid statement.

### 4.2. Tannin-Rich Forages Reduced Methane Formation Most, but Chicory Was the Best Species for Reducing Methane Concentration

One of our hypotheses in this study was that tannin-containing forages were able to reduce methane emissions more than non-tannin-containing forages. This was indeed the case for *S. minor* and *L. pedunculatus*, as both of these species showed the greatest methane reduction potential. However, both cultivars of *L. corniculatus* were less efficient at reducing methane than several of the tested non-tannin-containing forages. Nevertheless, within *L. corniculatus*, the ranking of the two cultivars was following the tannin concentrations, as Lotanava had greater tannin concentrations and also resulted in greater methane reductions than Leo.

Additionally, while the methane emissions were indeed reduced most by the species with the greatest tannin concentrations, this was different for the methane concentration. The methane concentration is an indicator for methane emissions per unit of digestible forage, and as such, is important for estimating whether or not methane reductions also decrease per unit of digestible forage. In this regard, *C. intybus* and *C. carvi* outperformed *S. minor* and *L. pedunculatus*, indicating that for the latter two species, there was a greater decrease in digestibility than methane, whereas *C. intybus* and *C. carvi* maintained high digestibility while also reducing methane. This is important, as *C. intybus* particularly is recognized as a species of high agronomic value for its dry matter yield potential and drought resistance and is frequently included in pasture-based systems [36,40], whereas *S. minor* and *L. pedunculatus* were outcompeted in the second sampling year of the present study and hence they could be sampled only once. The antimethanogenic activity of *C. intybus* and *C. carvi* could be explained by the presence of PSM other than tannins. The results were generally in agreement with values reported by Waghorn et al. [15] in an in vivo experiment, who found that sheep fed diets including chicory had lower methane emissions than those fed ryegrass–white clover and that these emissions were similar to those from sulla (*Hedysarum coronarium*), despite sulla containing PA concentrations of 3.5 to 6.8%. Similarly, Durmic et al. [41] reported that chicory and plantain reduced methane production by 25%, compared to *Trifolium vesiculosum*, in an in vitro batch-fermentation assay. Among the bioactive compounds present in chicory, sesquiterpene lactones and coumaric acid are of particular interest, as they have been shown to be beneficial for livestock production. The presence of sesquiterpene lactones in chicory also has been associated with anthelmintic properties. In an in vitro experiment carried out by Williams et al. [42], chicory extracts from two different cultivars were investigated for their anthelmintic effect against swine nematodes, and significant suppression of these parasites, when exposed to sesquiterpene lactones from chicory, was shown, although this effect was cultivar dependent. Anthelmintic effects in nematodes from lambs fed chicory have also been reported, for example, by Peña-Espinoza et al. [43].

The clover species *T. repens* and *T. pratense* are the most widely used legume forage species in European pasture systems [6]. Hence, the fact that they showed, on average, the lowest reductions in methane emissions and even increased methane concentration substantially compared to *L. perenne* is important. It is not very surprising, however, as neither of these species contains the important metabolites that have a role in reducing methane emissions. Regarding their metabolic profile, *T. repens* may contain PA, but only in the flowers [44]. As the flowers comprise only a small proportion of the forage biomass of *T. repens*, to obtain sufficient amounts of flowers to meet the PA requirement would likely require an excessive proportion of *T. repens* in the sward, with an associated risk of bloating [45]. Nevertheless, the PA contents of the samples harvested in 2018 suggest the presence of flowers in the samples, whereas the absence of PA in the samples from 2019 suggests that the sample comprised only leaves. This between-years difference in tannin composition could explain the greater methane reduction potential observed in 2018 (−9%) when compared to 2019 (−3%), as the slope of the latter was not significantly different from zero. For *T. pratense*, the major component that has been researched for its bioactivity is polyphenol oxidase (PPO). However, while PPO has been found to increase the nitrogen-use efficiency in silage making [46] and live-weight gains in ruminants [47], no benefits have previously been identified regarding methane emissions [48,49]. Consequently, due to the absence of bioactive substances and their concomitantly lower energy concentrations compared to *L. perenne* is a likely explanation for their increments in methane concentration.

### 4.3. Unlike Digestibility, Methane Formation Cannot Be Estimated Accurately Based on the Share and Type of Partner Species, or from Forage Quality Parameters Alone

While the total gas reduction showed comparably linear dose–effect responses with an increasing share of forage legumes or herbs, and the general methane reduction potential and improvements in methane concentration of all species was illustrated satisfactorily; estimating the methane formation of any particular plant species mixture remains difficult. Similarly, it was difficult to estimate the methane formation based on covariates that described forage quality or plant secondary metabolites. Regarding the forage quality covariates, this may be because the forage quality parameters were estimated based on NIRS. While these estimates were satisfactory for most partner species, the energy concentration and digestibility were overestimated compared to the estimates based on total gas for tannin-containing species. Consequently, this is likely to have affected their performance as covariables. The plant secondary metabolites, on the other hand, were very different, with almost no two species having comparable profiles, and this also makes their use as covariates difficult. Based on these factors, the estimation of methane formation from a distinct mixture of forage plant species remains difficult. Nevertheless, it remains unlikely that any minor addition of bioactive forb species is likely to achieve large benefits when applied at the field scale.

## 5. Conclusions

The present study has shown the potential of several forage species to reduce methane emissions from ruminants compared to diets based on highly digestible perennial ryegrass monocultures. The forbs salad burnet and big trefoil have shown substantial methane reduction potential. These were, however, accompanied by concomitant reductions in digestibility, as was evident by reduced total gas production. Accordingly, in terms of methane concentration, it was chicory rather than the species richest in tannins, which resulted in the lowest methane emissions per unit of digestible forage. This finding is of particular interest, as chicory also has greater agronomic potential as a component of sown forage mixtures for grazing. However, in contrast to the total gas production, methane production was less predictable, and linear increments in the legume or herb share did not result in linear changes in methane concentration but instead exhibited a comparably large variability. As a result, the extrapolation of findings to defined species mixture compositions for pasture swards remains difficult. Additionally, in vivo experiments with defined shares of the most promising species are required to identify whether these results can be extrapolated to the vastly more complex and diverse conditions in ruminants.

## Figures and Tables

**Table 1:** Plant species combined for the in vitro Hohenheim gas test (HGT), cultivar, common name, abbreviation, plant family and year of sampling.

	Species/Cultivar ^†^	Common Name	Abb *	Plant Family	Year
Common	*Lolium perenne*	Perennial ryegrass	*L. perenne*	Poaceae	2018, 2019
Partner	*Carum carvi*	Caraway	*C. carvi*	Apiaceae	2018, 2019
	*Cichorium intybus*	Chicory	*C. intybus*	Asteraceae	2018, 2019
	*Lotus corniculatus* “Leo”	Birdsfoot trefoil	*L. corniculatus* Leo	Fabaceae	2018, 2019
	*Lotus corniculatus* “Lotanava”	Birdsfoot trefoil	*L. corniculatus* Lotanava	Fabaceae	2018
	*Lotus pedunculatus*	Big trefoil	*L. pedunculatus*	Fabaceae	2018
	*Trifolium pratense*	Red clover	*T. pratense*	Fabaceae	2018, 2019
	*Trifolium repens*	White clover	*T. repens*	Fabaceae	2018, 2019
	*Plantago lanceolata*	Ribwort plantain	*P. lanceolata*	Plantaginaceae	2018, 2019
	*Sanguisorba minor*	Salad burnet	*S. minor*	Rosaceae	2018

* hereafter, species are referred to as abbreviations that are presented above; ^†^ except for *Lotus corniculatus*, the samples of each species harvested from the forage mixture is a combination of up to 4 cultivars in *L. perenne*, with most species consisting of 2 cultivars to manage the risk of unsuccessful establishment and ensure representativeness at the species level.

**Table 2:** Chemical composition and energy content of the species evaluated, harvested in 2018 and 2019.

Year	Species	ME_gas_	ME_NIRS_	NEL	NDF	ADF	DOM	CP
		(MJ/kg DM)			(g/kg DM)			
2018	*L. perenne*	11.6	10.5	6.5	508	271	850	108
	*C. carvi*	11.0	11.0	6.7	303	233	930	67
	*C. intybus*	11.1	11.3	6.7	346	236	920	100
	*L. corniculatus* Leo	11.2	11.2	6.8	351	217	840	178
	*L. corniculatus* Lotanava	10.7	10.9	6.6	375	240	830	182
	*L. pedunculatus*	9.6	10.3	6.1	449	290	800	173
	*T. pratense*	10.9	10.8	6.6	412	221	860	214
	*T. repens*	11.0	10.6	6.6	418	257	860	209
	*P. lanceolata*	11.1	11.3	6.9	341	232	910	133
	*S. minor*	10.6	12.2	7.0	417	253	890	149
2019	*L. perenne*	12.1	11.7	7.2	394	200	910	100
	*C. carvi*	10.2	10.3	6.3	414	291	870	145
	*C. intybus*	10.4	10.4	6.4	513	276	870	162
	*L. corniculatus* Leo	9.3	9.9	5.9	425	310	810	186
	*T. pratense*	NA	11.3	6.9	359	204	850	172
	*T. repens*	11.4	11.4	7.1	338	189	890	214
	*P. lanceolata*	9.9	9.8	6.1	582	310	790	169

**Table 3:** Tannin composition (mg/g DM) of the species evaluated, harvested in 2018 and 2019. (polyphenols (PP), flavonols (F), tannins (T), hydrolyzable tannins (HT), proanthocyanidins (PA), prodephinidins (PD)).

Year	Species	PP	F	T	HT	PA	PD Share
		(mg/g DM)					(%)
2018	*C. carvi*	8.8	4.1	0.0			
	*C. intybus*	9.3	2.4	0.0			
	*L. corniculatus* Leo	3.1	1.4	1.7	0.2	1.5	25
	*L. corniculatus* Lotanava	5.6	1.5	4.1	0.3	3.9	36
	*L. pedunculatus*	20.8	2.4	18.4	0.2	18.2	79
	*T. pratense*	4.5	4.0	0.3	0.0	0.3	44
	*T. repens*	6.1	4.0	1.8	0.0	1.8	100
	*P. lanceolata*	1.2	0.1	0.0			
	*S. minor*	47.6	5.6	41.0	41.0	0.0	0
2019	*C. carvi*	5.9	2.9	0.0			
	*C. intybus*	4.2	1.4	0.0			
	*L. corniculatus* Leo	3.6	1.3	2.0	0.1	1.9	30
	*T. pratense*	3.3	3.1	0.1	0.0	0.1	0
	*T. repens*	2.8	2.8	0.0			
	*P. lanceolata*	1.1	0.3	0.0			

**Table 4:** Mean values of measured in vitro fermentation parameters in a 24 h incubation period of species harvested in 2018 and 2019 consecutively. Numbers between brackets correspond to the standard error of the mean (SEM).

Species	Partner Prop.	Total Gas (mL/200 mg DOM ^1^)						Methane (mL/200 mg DOM)					
		2018			2019			2018			2019		
*L. perenne*	1	93.4		(3.23)	80.8		(2.38)	19.2		(1.68)	15.6		(0.24)
*C. carvi*	0.25	94.2		(0.68)	83.9		(0.68)	16.1		(0.07)	16.4		(0.32)
	0.375	89.5		(1.94)	82.4		(0.64)	16.5		(0.45)	16.2		(0.33)
	0.5	90.0		(0.72)	74.9		(0.41)	15.3		(0.34)	14.5		(0.16)
	0.675	86.8		(1.14)	82.2		(0.72)	14.3		(0.09)	16.1		(0.08)
	0.75	87.7		(1.22)	77.8		(0.64)	16.0		(0.43)	15.4		(0.45)
	1	83.8		(0.95)	77.3		(5.31)	16.6		(0.46)	15.1		(1.29)
*C. intybus*	0.25	94.3		(0.28)	81.2	^A^	(1.10)	16.0		(0.06)	15.9	^AB^	(0.28)
	0.375	86.2		(1.40)	81.3	^A^	(0.55)	14.4		(0.17)	16.2	^A^	(0.13)
	0.5	88.1		(1.57)	75.3	^AB^	(0.74)	15.4		(0.22)	14.4	^AB^	(0.07)
	0.675	86.2		(1.66)	80.9	^A^	(0.69)	13.6		(0.22)	15.9	^AB^	(0.09)
	0.75	84.8		(1.10)	75.5	^B^	(1.49)	15.3		(0.38)	14.7	^B^	(0.55)
	1	79.5		(0.69)	72.7	^AB^	(0.25)	15.7		(0.62)	14.0	^AB^	(0.15)
*L. corniculatus* Leo	0.25	91.4	^AB^	(2.25)	78.8	^AB^	(1.16)	16.7	^AB^	(0.24)	15.3	^A^	(0.19)
	0.375	88.0	^AC^	(1.34)	79.6	^A^	(0.50)	16.9	^A^	(0.41)	16.3	^AB^	(0.08)
	0.5	83.6	^CD^	(1.28)	76.7	^AB^	(1.00)	16.2	^AB^	(0.18)	14.9	^A^	(0.18)
	0.675	81.9	^ABCD^	(2.03)	75.7	^ABC^	(1.46)	14.7	^AB^	(0.17)	15.8	^AB^	(0.22)
	0.75	83.6	^jBD^	(1.05)	70.9	^kBC^	(0.73)	16.2	^B^	(0.44)	14.8	^AB^	(0.72)
	1	75.7	^BD^	(0.69)	63.8	^C^	(2.23)	16.5	^AB^	(0.37)	12.8	^B^	(0.23)
*T. pratense*	0.25	87.1	^A^	(1.84)	86.5		(0.87)	16.0	^A^	(0.34)	17.1	^A^	(0.28)
	0.375	82.8	^ABC^	(2.33)	87.6		(0.40)	16.5	^A^	(0.53)	17.5	^AB^	(0.37)
	0.5	82.9	^AB^	(0.72)	86.3		(1.07)	16.6	^AB^	(0.27)	17.5	^AB^	(0.34)
	0.675	80.2	^ABC^	(1.82)	89.4		(2.02)	14.9	^AB^	(0.15)	18.3	^B^	(0.35)
	0.75	80.1	^BC^	(3.20)	87.2		(2.06)	16.8	^B^	(0.35)	18.5	^B^	(0.08)
	1	72.0	^C^	(0.78)	82.8		(1.06)	16.7	^AB^	(0.62)	17.3	^B^	(0.20)
*T. repens*	0.25	96.0	^A^	(0.80)	81.4	^AB^	(0.67)	17.1		(0.03)	16.4		(0.37)
	0.375	82.9	^AB^	(1.62)	80.1	^AB^	(0.23)	16.5		(0.44)	16.4		(0.41)
	0.5	89.1	^ABC^	(0.55)	75.6	^AC^	(0.44)	16.7		(0.15)	15.0		(0.43)
	0.675	81.5	^ABC^	(0.69)	79.3	^AB^	(1.04)	15.3		(0.08)	16.6		(0.22)
	0.75	77.9	^C^	(1.16)	73.5	^CD^	(1.19)	16.3		(0.51)	16.3		(0.41)
	1	75.4	^BC^	(0.90)	69.8	^BD^	(0.20)	17.5		(0.55)	15.1		(0.04)
*P. lanceolata*	0.25	95.3	^A^	(0.95)	82.4		(1.83)	16.4	^ABCD^	(0.11)	16.2		(0.57)
	0.375	87.0	^AB^	(0.84)	79.6		(0.82)	15.2	^AB^	(0.10)	15.8		(0.20)
	0.5	87.5	^A^	(0.82)	82.6		(0.53)	16.5	^AC^	(0.23)	16.4		(0.09)
	0.675	83.9	^AB^	(1.54)	79.5		(1.14)	14.3	^CD^	(0.24)	15.9		(0.36)
	0.75	81.2	^AB^	(1.40)	77.5		(2.36)	15.4	^ABCD^	(0.43)	15.7		(0.45)
	1	77.3	^B^	(1.50)	72.6		(4.55)	16.1	^BD^	(0.37)	13.7		(0.75)

^1^ DOM: digestible OM; ^j,k^ noncommon lowercase letters indicate differences between years; ^A–D^ noncommon uppercase letters indicate differences between partner proportions.

**Table 5:** Mean values of measured in vitro fermentation parameters in a 24 h incubation period of *Lotus corniculatus cv.* Lotanava*, Lotus pedunculatus*, and *Sanguisorba minor* samples, harvested in 2018.

Species	Partner Prop.	Total Gas			Methane		
		(mL/200 mg DOM ^1^)					
*L. corniculatus* Lotanava	0.25	90.7	^AB^	(2.70)	16.3	^AB^	(0.42)
	0.375	87.8	^A^	(1.59)	16.7	^A^	(0.42)
	0.5	81.7	^BC^	(1.18)	15.6	^AB^	(0.35)
	0.675	82.7	^ABC^	(1.51)	14.6	^AB^	(0.11)
	0.75	80.7	^BC^	(1.29)	15.7	^B^	(0.46)
	1	71.9	^C^	(2.46)	15.8	^AB^	(0.47)
*L. pedunculatus*	0.25	87.7	^AB^	(3.70)	15.5	^AB^	(0.56)
	0.375	82.1	^AC^	(0.96)	14.1	^A^	(0.06)
	0.5	79.7	^ABCD^	(0.29)	14.9	^AB^	(0.06)
	0.675	73.4	^BD^	(3.14)	12.5	^B^	(0.25)
	0.75	71.7	^CD^	(1.07)	13.7	^AB^	(0.38)
	1	63.6	^D^	(1.59)	13.3	^AB^	(0.52)
*S. minor*	0.25	89.3	^A^	(1.17)	15.4	^ABCD^	(0.21)
	0.375	79.8	^A^	(1.58)	14.5	^AB^	(0.38)
	0.5	79.5	^AB^	(1.96)	15.0	^AC^	(0.44)
	0.675	72.8	^ABC^	(2.32)	11.9	^BD^	(0.14)
	0.75	71.8	^BC^	(1.15)	13.0	^CD^	(0.34)
	1	63.3	^C^	(1.31)	13.4	^BD^	(0.68)

^1^ DOM: digestible OM; ^A–D^ noncommon uppercase letters indicate differences between partner proportions.

**Table 6:** Mean adjusted R^2^ and *p* values derived from basic linear regression for methane production (mL/200 mg DOM), total gas production (mL/200 mg DOM) and methane concentration after 24 h-rumen fermentation of the studied forage species harvested in 2018 and 2019. (Significance levels are: *** *p* ≤ 0.001, ** *p* ≤ 0.01, * *p* ≤ 0.05 and ns *p* > 0.05).

Year	Species	Total Gas		Methane		Methane/Total Gas	
		Adjusted R^2^					
2018	*C. carvi*	0.19	**	−0.02	ns	0.12	*
	*C. intybus*	0.49	***	0.00	ns	0.31	***
	*L. corniculatus* Leo	0.52	***	0.00	ns	0.26	***
	*L. corniculatus* Lotanava	0.48	***	0.03	ns	0.28	***
	*L. pedunculatus*	0.72	***	0.12	*	0.46	***
	*T. pratense*	0.33	***	−0.02	ns	0.48	***
	*T. repens*	0.46	***	−0.02	ns	0.47	***
	*P. lanceolata*	0.59	***	0.00	ns	0.39	***
	*S. minor*	0.74	***	0.22	**	0.20	**
2019	*C. carvi*	0.23	**	0.09	ns	−0.03	ns
	*C. intybus*	0.40	***	0.34	***	0.01	ns
	*L. corniculatus* Leo	0.74	***	0.39	***	0.00	ns
	*T. pratense*	−0.01	ns	0.11	ns	0.23	**
	*T. repens*	0.69	***	0.02	ns	0.27	**
	*P. lanceolata*	0.25	**	0.17	**	−0.03	ns

## Data Availability

The data presented in this study are available on request from the corresponding author.

## Appendix A

**Table A1:** Statistical data of the NIRS calibration and validation (SEC, standard error of calibration; SEV, standard error of prediction) for the relevant quality parameters by plant group.

Parameter	Plant Group	N	Mean	Range	SEC	R^2^	SEP
ME (MJ/kg DM)	Whole sward	251	10.810	8.38–12.62	0.179	0.959	0.195
ME (MJ/kg DM)	Grasses	248	10.806	8.38–12.62	0.173	0.956	0.194
	Legumes	168	10.737	8.55–12.41	0.15	0.961	0.196
	Herbs	117	10.691	8.38–12.54	0.154	0.963	0.211
NEL (MJ/kg DM)	Whole sward	249	6.567	4.79–7.81	0.138	0.955	0.173
NEL (MJ/kg DM)	Grasses	250	6.569	4.79–7.87	0.136	0.949	0.193
	Legumes	167	6.512	4.92–7.77	0.106	0.965	0.152
	Herbs	115	6.514	4.80–7.784	0.112	0.964	0.163
DOM (g/kg DM)	Whole sward	249	809.45	642.6–919.0	8.85	0.967	9.14
DOM (g/kg DM)	Grasses	248	821.04	632.1–918.8	10.83	0.956	10.97
	Legumes	173	803.63	657.6–902.6	10.36	0.944	12.96
	Herbs	118	817.42	642.6–904.3	13.51	0.943	12.45
N (g/kg DM)	Whole sward	268	26.69	8.7–54.2	0.951	0.991	1.071
N (g/kg DM)	Grasses	277	22.59	9–54.9	0.802	0.991	0.914
	Legumes	178	35.07	14.7–57.1	1.116	0.981	1.131
	Herbs	86	26.39	10.3–39.9	0.749	0.995	1.317
